# Antibacterial Properties of Silver Nanoclusters with Carbon Support on Flexible Polymer

**DOI:** 10.3390/nano12152658

**Published:** 2022-08-03

**Authors:** Klaudia Hurtuková, Tereza Vašinová, Nikola Slepičková Kasálková, Dominik Fajstavr, Silvie Rimpelová, Vladimíra Svobodová Pavlíčková, Václav Švorčík, Petr Slepička

**Affiliations:** 1 Department of Solid State Engineering, University of Chemistry and Technology Prague, 166 28 Prague, Czech Republic; klaudia.hurtukova@vscht.cz (K.H.); tereza.vasinova@vscht.cz (T.V.); nikola.kasalkova@vscht.cz (N.S.K.); dominik.fajstavr@vscht.cz (D.F.); vaclav.svorcik@vscht.cz (V.Š.); 2 Department of Biochemistry and Microbiology, University of Chemistry and Technology Prague, 166 28 Prague, Czech Republic; silvie.rimpelova@vscht.cz (S.R.); vladimira.svobodova.pavlickova@vscht.cz (V.S.P.)

**Keywords:** carbon, excimer laser, antibacterial properties, silver nanoclusters, plasmon resonance, nanostructure

## Abstract

Here, we aimed at the preparation of an antibacterial surface on a flexible polydimethylsiloxane substrate. The polydimethylsiloxane surface was sputtered with silver, deposited with carbon, heat treated and exposed to excimer laser, and the combinations of these steps were studied. Our main aim was to find the combination of techniques applicable both against Gram-positive and Gram-negative bacteria. The surface morphology of the structures was determined by atomic force microscopy and scanning electron microscopy. Changes in surface chemistry were conducted by application of X-ray photoelectron spectroscopy and energy dispersive spectroscopy. The changes in surface wettability were characterized by surface free energy determination. The heat treatment was also applied to selected samples to study the influence of the process on layer stability and formation of PDMS-Ag or PDMS-C-Ag composite layer. Plasmon resonance effect was determined for as-sputtered and heat-treated Ag on polydimethylsiloxane. The heating of such structures may induce formation of a pattern with a surface plasmon resonance effect, which may also significantly affect the antibacterial activity. We have implemented sputtering of the carbon base layer in combination with excimer laser exposure of PDMS/C/Ag to modify its properties. We have confirmed that deposition of primary carbon layer on PDMS, followed by sputtering of silver combined with subsequent heat treatment and activation of such surface with excimer laser, led to the formation of a surface with strong antibacterial properties against two bacterial strains of *S. epidermidis* and *E. coli*.

## 1. Introduction

Any material with particle dimensions ranging from 1 to 100 nm in at least one dimension can be marked as a nanomaterial. According to the shape of nanomaterials, they are divided into three groups: 0D (nanoparticles, fullerenes, etc.), 1D (nanotubes, nanofibers, etc.), and 2D (nanolayers, e.g., graphene) [1]. Nanomaterials have recently gained great popularity since they exhibit significantly distinct properties from their macroscopic forms [2], which is caused by their higher surface-to-volume ratio. For the macroscopic forms of materials, we can omit the effect of surface atoms interacting with the surrounding environment, but this does not hold for nanomaterials. As a result, nanomaterials have different mechanical properties, such as strength, toughness, or elasticity. Moreover, they also often exhibit different electrical and thermal conductivities [3,4,5]. An important nanomaterial from carbon is graphene, where the atoms are connected in one plane into a honeycomb shape, thus forming a planar structure with a width of one atom [6,7]. Graphene is considered as the thinnest material in the world [7,8]. It is a relatively new material, first synthesized in 2004 [7,8,9,10]. It is remarkable mainly due to its electronic properties. Electrons in graphene interact with the periodic potential of the structure, leading to the formation of new quasiparticles. Graphene applications have a large potential in numerous fields, such as electronics and medicine [8,11,12].

As aforementioned, nanomaterials offer many advantages over their classical macroscopic forms. They also suffer from certain disadvantages such as the lack of stability and the tendency to aggregate. These limitations can, however, be solved by the preparation of a composite formed by nanoparticles and a polymer matrix [13]. Such composites consist of two or more phases that substantially differ in their properties. They usually consist of a matrix (continuous part) and reinforcement. The reinforcement should improve the mechanical properties of the matrix and, therefore, tends to have a higher modulus of elasticity, strength, etc. [14]. The properties of the composite are significantly improved, rather than just the sum of the properties of the individual components [15]. Reinforcements are of two types: fibers or particles [15,16]. Due to excellent thermal and chemical stabilities and good dielectric properties, polydimethylsiloxane (PDMS) is widely used in the automotive industry as a sealant or seal; it can also be found in radiators, engine covers, valves, etc. [17]. Since PDMS is resistant to oxidation, it can be also utilized in metal coatings to prevent corrosion and fouling [18]. Further, high PDMS hydrophobicity is commonly used in waterproof materials, for self-cleaning, and as an antifreeze [19]. Moreover, owing to PDMS biocompatibility and inertness, it can be also applied for soft tissue implants or heart valve replacements [20]. This material can be also used in the production of contact lenses intended to treat permanent corneal damage. Human epithelial cells grow well on PDMS materials, and therefore can be inoculated directly onto the polymer components, thus, speeding up the treatment [21]. PDMS can be also found in soft tissues in the manufacture of nanorobots [22], and in combination with nanoparticles.

Precious metal nanoparticles (especially gold, silver, and platinum) are widely used in various industries due to their unique properties. It is important to note that nanoparticle properties depend on their shape (whether spherical or with edges) and their size [23]. They can be used as catalysts, photocatalysts, or sensors [24]. Gold nanoparticles are very stable, non-reactive, and biocompatible. Gold forms strong bonds with thiol groups, which enables the binding of specific thermosensitive proteins to the carrier surface, and their use as drug carriers. Due to the proteins, a controlled drug release occurs upon temperature changes [25,26]. A similar principle can be used when binding pH-sensitive molecules are used in the same manner [27]. On the contrary, silver nanoparticles have excellent bactericidal properties, as a result of which they are used in food storage containers, socks, sportswear, water purification filters, etc. [28]. Silver nanoparticles can also be used to treat dental diseases. Due to the still-growing bacterial resistance to existing antibiotics, silver nanoparticles could partially replace them. Nevertheless, the high cytotoxicity of silver nanoparticles in mammalian cells also represents a significant problem, especially for medical applications.

The noble metals have also been studied in the form of thin films, where metal deposition may occur at higher amounts or with long-term use, resulting in skin breakage or discoloration [29]. Applications of thin metal films to solid substrates are being investigated, since unique materials can be created. There have been various techniques, such as Langmuir-Blodgett (preparation of single-molecule layers), lithographic methods, sputtering, or pulsed laser deposition (PLD) [30]. The sputtering method is popular for applying a wide range of coatings. Chemical approaches for preparation and application of activated silver clusters have been studied in [31]. Thus, wear- or corrosion-resistant layers or specific optical or electrical properties can be prepared [32]. This is advantageous mainly due to the uniform application of metal on the substrate, but is also relatively fast and environmentally friendly; everything is carried out at laboratory temperatures and is suitable for several types of polymeric materials [33]. Bacteria occur either as planktonic, i.e., living as separate units, or they form a biofilm. The biofilm consists of immobile cells that are trapped in a matrix of organic origin. Bacteria found in the biofilm typically do not perform Brownian motion and have a spongy shape. To create a biofilm, two key ingredients are necessary: bacteria and a substrate.

If there is a shortage of water in the environment, bacterial mobility, as well as nutrient availability, decreases. In such conditions, planktonic bacteria do not survive, unless they create a biofilm, which can be beneficial for bacteria in many ways. For instance, in a biofilm, bacteria can be protected from antibiotics, disinfectants, or rapid changes in environmental conditions. Moreover, bacteria in the biofilm can communicate with the matrix and thus promptly regulate gene expression, which enables their fast adaptation to different conditions [34]. Different bacterial adhesion can be observed, for example, to materials with different charges. Most bacteria have a negative charge, so they prefer to stick to a positively charged surface. However, there are cationic groups that, in turn, have a bactericidal effect. In static systems, a barrier from dead cells is formed on the surface, and new bacteria do not tend to adhere to the material [35]. This also depends on the surface wettability. Hydrophilic materials can resist bacterial adhesion, while hydrophobic materials are frequently colonized. In terms of roughness, rougher materials have a larger surface area and are more suitable for bacterial colonization, while reduced roughness may positively influence antibacterial properties [36].

In this study, we focused on the preparation of silver nanostructures on a flexible PDMS substrate. The heat treatment was further applied to study the influence of the process on layer stability and properties. The surface plasmon resonance effect was determined for an as-sputtered and heat-treated system. Based on our previous research, we have also implemented the sputtering of the carbon base layer in combination with excimer laser exposure. The main novelty of this paper is based on application of carbon base layer followed by silver nanolayer sputtering on PDMS, which in combination with subsequent heat treatment and high energy excimer exposure led to the formation of a surface with strong antibacterial properties against both Gram-positive and Gram-negative bacteria. Even if the antibacterial effect of Ag is well known, we believe that such an approach may contribute to this field of research.

## 2. Materials and Methods

### 2.1. Materials

In this study, polydimethylsiloxane (PDMS, with a density of 1.5 g·cm^−3^) in the form of 50 μm thick sheets (supplied by Goodfellow, Ltd., Huntingdon, UK) was used as a substrate for the performed experiments.

The carbon layers were deposited using an SDC 050 Carbon Thread Evaporation Device by the flash evaporation process, using a carbon fiber (Leica). The carbon filament was degassed at a current of 1.5 A (pressure 4 Pa). The polymer substrate was at a distance of 5 cm from the carbon fiber. Corresponding thicknesses of C layers were determined on a quartz glass substrate with a scratch method and were with an atomic force microscope. The distance of 5 cm represents the thickness of 8.1 ± 0.7 nm.

Sputtering of thin Ag layers on the surfaces of pristine PDMS and PDMS with a C layer was performed using the Quorum Q300T ES with cathode sputtering. Different sputtering times of 50, 100, 200, 300, 400, and 1000 s and sputtering current of 10, 20, 40, and 80 mA were used. A set of samples was prepared to determine the thicknesses (20 nm) of the sputtered thin Ag layers using the scratch test method on Si substrate. A silver target (with a purity of 99.999%, purchased from Safina, Vestec, The Czech Republic) was used to prepare the nanolayers. Thermal treatment of pristine and silver-coated polymer was performed at the upper working temperature of PDMS, i.e., 285 °C, for 1 h in a Binder oven with a thermostat. Teflon foil was added as a primary substrate. Teflon was chosen since it withstands the chosen high temperature and, at the same time, it is non-stickable. Therefore, it prevented the PDMS foils from adhering to the glass surface of Petri dishes, which occurred at such high temperatures. The annealed samples were cooled in the air at room temperature (25 °C).

### 2.2. Analytical Methods

The measurement of contact angles (goniometry) was performed using a goniometer Advex Instruments (Brno, Czech Republic), which was connected to the SEE System 7.1 program. A drop of distilled water and glycerol with a volume of 8 µL was added dropwise to the sample using a Transferpette^®^ automatic pipette (Brand, Wertheim, Germany). Subsequently, the drop was photographed using 3 marked points, and the program evaluated the line that delimits the drop and the corresponding contact angle. Finally, the surface free energy was determined by Owens-Wendt method.

The Dimension ICON atomic force microscope (Bruker, Billerica, MA, USA) and in ScanAsyst^®^ mode were used to detail the surface morphology of PDMS and PDMS/Ag films. The sharp silicon tip was mounted on a SCANASYST-AIR nitride arm with a spring constant of 0.4 N·m^−1^. The measured data were then processed using NanoScope^®^ Analysis software, the size of the resulting images being 1 × 1 μm^2^. R_a_ (surface roughness) represents the arithmetic mean of the absolute values of the height deviations measured from the central plane.

The morphology of the samples was also measured and analyzed by a FIB-SEM LYRA3 Tescan scanning electron microscope, for which the samples had to be pre-plated (platinum, 10 nm) to ensure their conductivity.

Surface chemistry was characterized using an Omicron Nanotechnology ESCAProbeP X-ray photoelectron spectrometer. Monochromatic X-rays with an energy of 1486.7 eV were used as the source. An area of 2 × 3 mm^2^ was analyzed.

Absorption spectra were measured using a PerkinElmer instrument, Lambda 25 spectrometer type. The spectrometer has a wavelength range of 190–1100 nm is suitable for both solutions and solid samples.

### 2.3. Antibacterial Tests

Gram-negative and Gram-positive bacterial strains of *E. coli* (DBM 3138) and *S. epidermidis* (DBM 2124), respectively, were used for the evaluation of antibacterial activity of the prepared materials. The bacterial strains were inoculated from agar plates into Luria–Bertani (LB) medium and subsequently cultured overnight at 37 °C in an orbital shaker. The optical densities of the bacterial cultures were measured at 600 nm and serially diluted. The number of 2·10^4^ and 4·10^4^ of colony forming units (CFU) of *E. coli* and *S. epidermidis*, respectively, were inoculated per 1 mL of sterile phosphate buffered-saline (PBS, pH of 7.4) into which the test samples were immersed. The samples (same sizes for all experiments) were then mixed gently and incubated dynamically at 24 °C for 1 and 4 h. The samples were then gently mixed again, and 20 μL drops of each sample (3 biological replicates) were pipetted onto LB agar plates (*E. coli*, *S. epidermidis*). The plates were then cultured at 37 °C for 24 h, after which the number of CFUs was counted and compared with the number of CFUs on control plates (bacteria incubated only in PBS without any samples added, at the same time points).

## 3. Results

### 3.1. Surface Morphology: AFM and SEM Methods

Our research was aimed at the preparation of PDMS samples deposited with silver nanolayer and/or pre-deposited with carbon, which were subsequently modified with heat treatment or excimer laser, and to study the surface and antibacterial properties. The PDMS films were exposed to different currents (20, 40, and 60 mA) and time (50 and 400 s) of silver sputtering.

**Figure 1:**
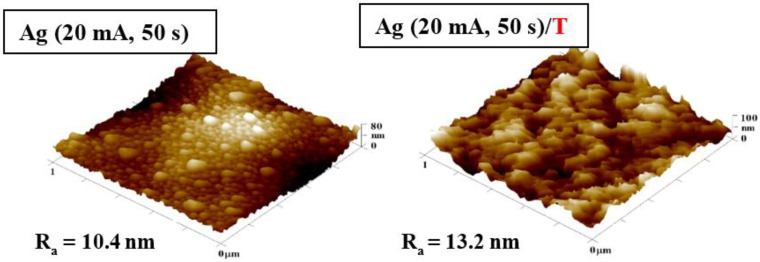
Atomic force microscopy images of PDMS samples deposited with Ag 20 mA and 50 s, and same sample subsequently heated at 285 °C. The inspected area was 1 × 1 µm^2^. R_a_ represents the average roughness in nm.

Figure 1 compares AFM images of PDMS films with an Ag layer sputtered at 20 mA for 50 s before and after thermal treatment. As seen in Figure 1, the application of 285 °C changed the morphology and surface roughness of the PDMS films. Before the heat treatment, the samples exhibited a nanocluster-like Ag structure on the PDMS surface; however, after 1 h in the oven, the structure diminished. This phenomenon was caused by the set temperature, which reached values higher than the upper working temperature of PDMS. The thermal stress also increased the material surface roughness from 10.4 nm to 13.2 nm.

**Figure 2:**
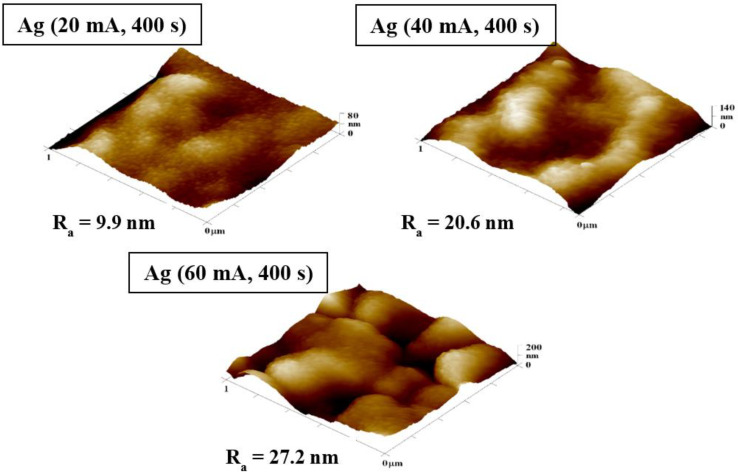
Atomic force microscopy images of PDMS samples deposited with Ag nanoparticles for 400 s at different currents of 20, 40, and 60 mA. The inspected area was 1 × 1 µm^2^. R_a_ represents the average roughness in nm.

On the other hand, Figure 2 compares AFM images of samples with Ag layers sputtered for 400 s and different values of current: 20, 40, and 60 mA. For samples modified at higher current (40 and 60 mA), we have observed a change in the surface structure and an increasing roughness. When comparing samples modified at 20 mA and 60 mA, it should be noted that the higher current created a structure induced by a more substantial bombardment of Ag nanoparticles on the sample surface, and therefore partial “disruption” of the Ag layer takes place (Figure 2, bottom image).

**Figure 3:**
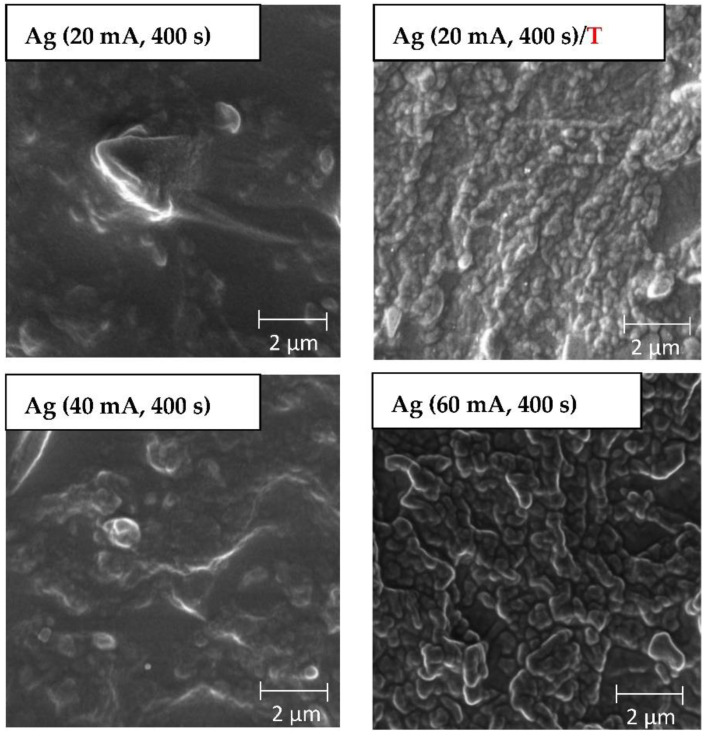
Scanning electron microscopy images of PDMS samples deposited with Ag for 400 s currents of 20 mA and subsequent heating at 285 °C, 40 mA, and 60 mA. The inspected area was 10 × 10 µm^2^.

The SEM method was chosen as another method to study the surface morphology of the prepared samples. It was decided to analyze the larger surface area of samples with an Ag layer sputtered for 400 s at the current of 20 mA, and 20 mA followed by thermal treatment at 285 °C, 40 mA, and 60 mA (see Figure 3). We applied the heat treatment only for the layer with shortest deposition time, since on the basis also of our previous preliminary experiments in combination with carbon pre-deposition, it seemed to be “the best” candidate from the point of surface effective area and morphology change. The created patterns on a larger scale confirm the results from the AFM analysis. In Figure 3, a change in the surface roughness of the unheated samples was observed, mainly due to the deposition of a thicker Ag layer. For samples modified at 60 mA for 400 s, the change in surface morphology was the most significant. The wrinkle-like pattern formation was induced by the presence of a surface bilayer enhanced by silver nanoparticles. The bilayer exhibited different parameters regarding heat conductivity, and thus, during the cooling process, this inhomogeneity induced the pattern formation.

### 3.2. Surface Chemistry Analysis

**Figure 4:**
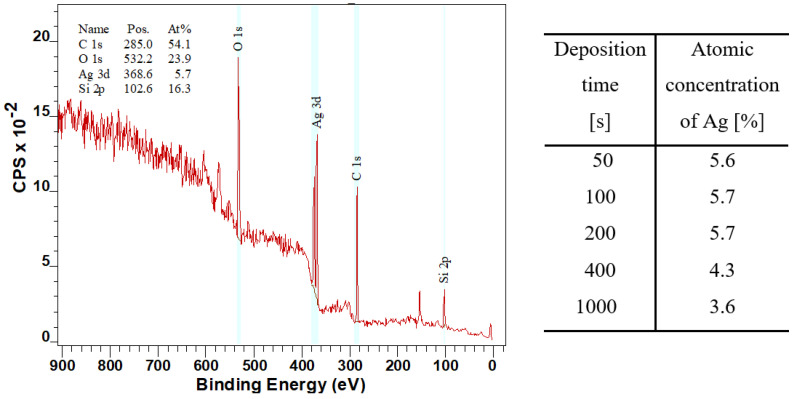
XPS spectrum for a sample deposited at 20 mA for 200 s and a table with the atomic concentration of Ag in prepared samples at a current of 20 mA with different sputtering times (50, 100, 200, 400, and 1000 s).

The changes in the chemical composition of the surface were studied using the XPS method, which under normal conditions measures only to a depth of 10 nm [37]. As an alternative elemental analysis, we have applied the EDS method to acquire elemental composition data from a larger depth, since EDS analysis enables the determination up to a depth of several hundreds of nanometers [38]. Figure 4 shows the XPS spectrum of a sample deposited with an Ag layer at 20 mA for 200 s and a table with the atomic concentration of Ag for samples sputtered at a current of 20 mA at different deposition times. According to the table in Figure 4, the value of atomic concentration decreased with increasing deposition time, which was not expected and came into conflict with the expectations. Even though this experiment was conducted repeatedly several times, we came to similar values and conclusions. The explanation for the change in PDMS surface composition (Ag concentration) could be therefore related to the partial implantation of Ag atoms into the polymer volume. Another explanation is the dramatic change in surface morphology for high-dose samples, for which a wavy PDMS/Ag composite formation occurred, and a not completely homogeneous distribution of Ag atoms within the nanostructure was therefore observed. These unexpected results implicate the use of lower sputtering doses for PDMS metallization.

**Figure 5:**
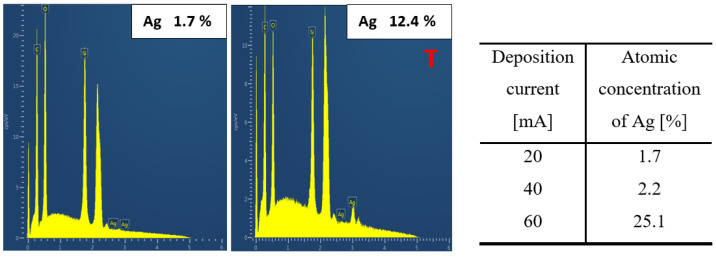
EDS spectrum of a sample deposited at 20 mA for 400 s, unheated 20 mA and 400 (**left**) and heated 20 mA and 400 (**right**), and a table with the atomic concentration of Ag of the prepared samples at a sputtering time of 400 s at different currents (20, 40, and 60 mA).

To partially confirm this assumption, the chemical composition was also measured by EDS analysis, which is less surface sensitive than XPS—meaning detailed surface analysis and analysis of lighter elements, such are oxygen and carbon. Here, we introduce the EDS analysis results, both selected spectra and particular atomic concentrations (see Figure 5).

The atomic proportion of Ag for the heated sample was higher than for the unheated sample (see Figure 5). When comparing the effect of heat treatment, it is evident that there was a greater penetration of nanoparticles into the volume of the polymer during thermal treatment, thus the composite formation is expected. The diffusion in combination with wrinkle pattern formation probably leads to an increase in atomic concentration of Ag, as described for non-heated and heated sample in Figure 5. When the deposition time was maintained and the deposition current increased, the atomic concentration of Ag increased, as shown in the table in Figure 5. This phenomenon partially explains the results acquired with XPS analysis, since the decrease in silver concentration was not confirmed with EDS. We have also determined the wettability of as-sputtered and heated samples. As is obvious from Table 1, silver sputtering leads surprisingly to a slight decrease of surface energy, and the material is less wettable. For the thin Ag layers the heat treatment (1 h, 285 °C) leads to wettability decrease (50 s), or increase (Ag layer deposited 1000 s), or the changes are only minor compared to non-heated samples.

### 3.3. Optical Properties

**Figure 6:**
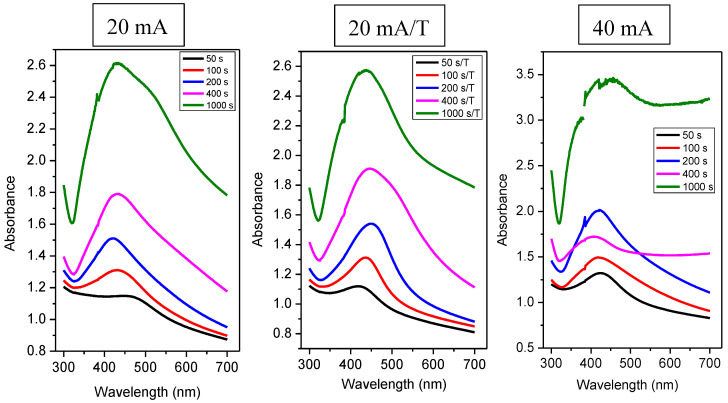
UV-VIS spectrum of Ag layers on PDMS—a comparison of different deposition times at constant current (20 and 40 mA) or with the following thermal treatment (20 mA/T).

One of the phenomena which may be observed in metal nanoparticle systems is the effect of surface plasmon resonance, which can be confirmed by the UV-VIS absorption technique. The shape of the absorption peak, its position, and other specific properties of the Ag spectra depend primarily on the size of silver nanoparticles and their number [39]. The SPR effect can be observed mainly in colloidal systems but it can also be identified on the surface of solid samples. It was found that the absorbance maximum increases with the increasing number of Ag nanoparticles on the PDMS surface (see Figure 6). Further, it is also obvious that the samples showed a peak of plasmon surface resonance for different doses of Ag atoms, which was preserved also after thermal annealing; the position of the peak is typical for Ag nanoparticles (more likely clusters in our case). The thermal treatment of PDMS/Ag induced a slight shift of absorption maxima to larger wavelengths (20 mA/T), and both heated and unheated samples sputtered for 1000 s exhibited a significant increase in the absorbance peak width, which was caused by coalescence of Ag nanoparticles into either larger agglomerates or a continuous layer.

This phenomenon is the same for all samples, except for a sample with deposition at 40 mA for 400 s, which had a lower absorbance compared to sample modified at 40 mA for 200 s, but the peak of surface plasmon resonance was preserved. For silver nanoparticles, the value of the absorption maximum in solution at wavelengths around 400 nm is typical [39]. The position of the absorbance maxima of the measured spectra was always at wavelengths slightly higher than 400 nm (see Table 2). According to Petit et al. [40], for small particles (≤30 Å), the position of the absorption maximum shifts, but the peak width of the surrounding medium is also changed. When the particles interact with the matrix only via weak interactions, there was a bathochromic shift (a redshift) and a slight broadening of the peak. On the contrary, with strong interactions, there was a hypso-chromic shift (a blue shift) [40] and a significant peak broadening.

**Figure 7:**
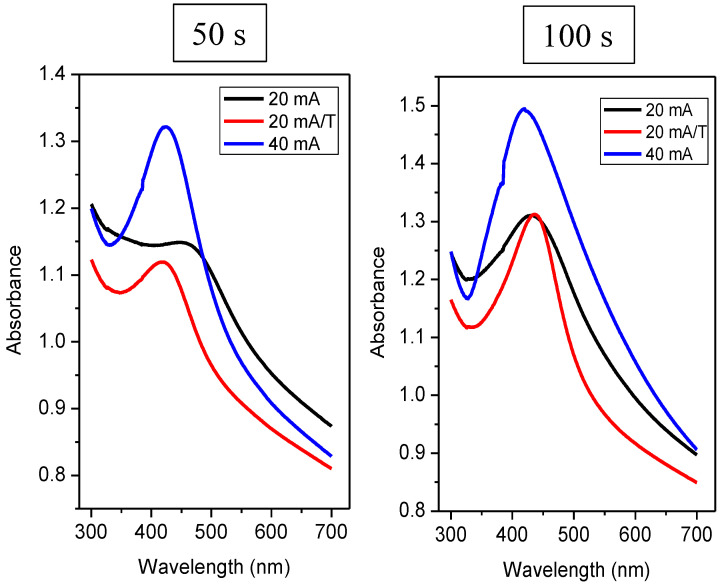
UV-VIS spectra of Ag layers on PDMS at different deposition times of 50 and 100 s, and currents of 20 and 40 mA or samples with the subsequent thermal treatment (20 mA/T).

The bathochromic shift of the absorption maxima can be explained also by the influence of the dielectric layer, where the formation of PDMS-Ag composite may have the same effect as observed for tunable dipole surface plasmon resonances systems [41], the dielectric constant PDMS being in the range of 2.58–2.70 [42]. Generally, a larger peak width also indicates greater dispersion of particle sizes and shapes [43]. The curve shape can also tell us what the shape of the nanoparticles is. Our values can assume that the particles are spherical, as the peak shows only one absorption maximum. Figure 7 shows the spectrum of samples with a deposition duration of 50 s and 100 s.

**Figure 8:**
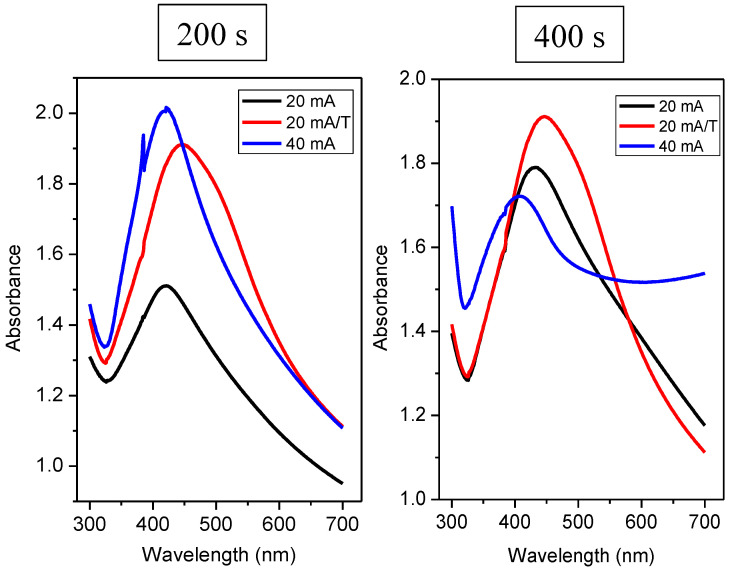
UV-VIS spectrum of Ag layers on PDMS at different deposition times 200 s, 400 s, and current of 20 and 40 mA or samples with the subsequent thermal treatment (20 mA/T).

Comparing heated and unheated samples with a deposition time of 50 s (Figure 7), we found that the unheated sample at 20 mA did not exhibit a strong plasmon peak (see black curve on the left in Figure 7). At the same time, the absorption maximum was reached at 448 nm. From this significant shift and shape of the curve compared to other samples deposited at 20 mA, we suggest that this combination of current and deposition time was not sufficient for the formation of isolated spherical nanoclusters, so even at low currents, we saw that probably insufficiently large nanoparticles inhomogeneously distributed on the surface led to a change in the UV-VIS response. The curve for the same sample, only thermally stressed, already had the characteristic shape typical for a nanoparticle system. Therefore, upon heating, nanoparticles/nanostructures formed on the PDMS substrate. We can also observe a sharp peak in the sample deposited at a twofold current (i.e., 40 mA), which confirms the presence of isolated nanoparticles. From Figure 7, it is also clear that when we deposited silver at a current of 20 mA and the time was increased to 100 s, this combination of the deposition time and current was enough to form nanoparticles. Regarding the absorbance of heated and unheated samples (i.e., at 20 mA), we observed a similar value for times 50 and 100 s. However, for times of 200 and 400 s, the absorbance increased significantly compared to unheated samples as expected (see Figure 8).

Table 2 contains the evaluated absorption maxima and their corresponding wavelengths. It can be said that the absorption peak’s maxima for the heated samples were shifted slightly towards higher wavelengths, i.e., radiation of lower energy. This trend applies to all samples, except for samples deposited for 50 s—a combination of 20 mA and 50 s was not suitable to form sufficiently isolated large nanoparticles. It was noted that the bathochromic shift of absorption peaks was characteristic of an increase in nanoparticles’ dimension. When heated, the particles migrated along the surface and agglomerated. When we compare the absorption spectra of layers deposited at currents of 20 and 40 mA, at higher currents, there was a hypso-chromatic shift of their absorption maxima. A smaller or larger “tooth” was formed during all measurements, each time in the same area, i.e., around 390 nm. The position of this “step” was sample-independent, and this anomaly is related to the UV-VIS measuring instrument.

### 3.4. Antibacterial Properties

Based on our previous experiments we have chosen a selection of samples for antibacterial properties. Due to previous research, we have also implemented the sputtering of the carbon base layer in combination with excimer laser exposure. The formation of PDMS-noble metal nanocomposite and noble metal nanostructures revealed interesting properties for SERS analysis [44]. The heating of such structures resulted in formation of pattern which exhibited surface plasmon resonance effect [45,46], the effect which may also influence the antibacterial results significantly. On the contrary, carbon nanolayers exhibit interesting biocompatible properties, either in a form of isolated clusters or layers [47,48,49].

Here, the antibacterial properties were investigated on selected PDMS samples with an Ag layer sputtered for 300 s at different currents of 10 mA (Ag 10 mA, 300 s) 40 mA (Ag 40 mA, 300 s), and 80 mA (Ag 80 mA, 300 s). They were compared with pristine PDMS (pristine) and PDMS samples, whereas the base layer deposited a carbon nanolayer. Several combinations were also tested. PDMS sample was coated with carbon and Ag layer sputtered at a current of 40 mA and deposition time of 300 s (C/Ag), the same combination of layers subsequently heated at 285 °C for 1 h (C/Ag/T), and the heated system followed by surface modification with a single excimer laser shot with fluence 150 mJ·cm^−2^ (C/Ag/T/L).

**Figure 9:**
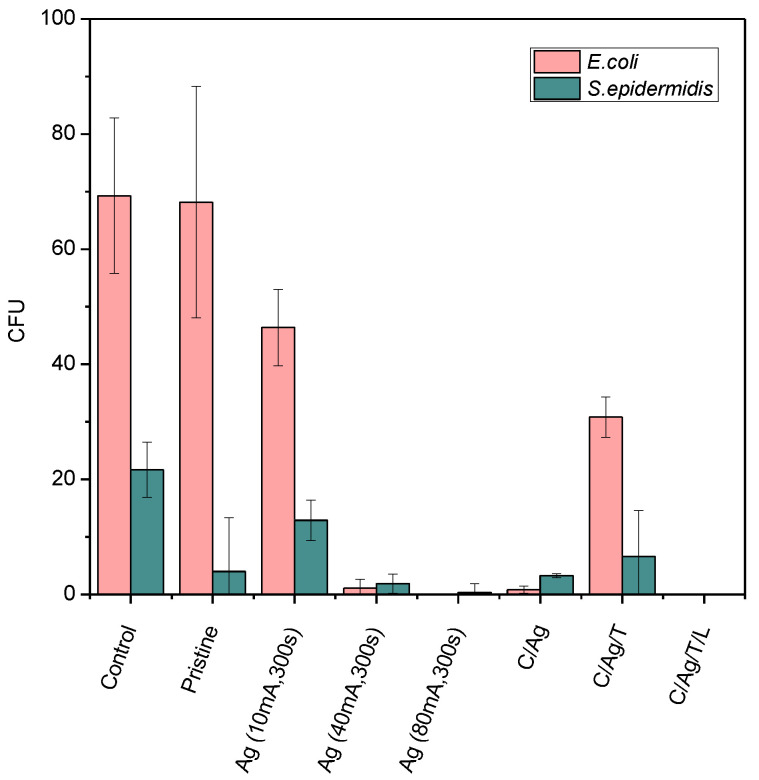
The number of colony-forming units (CFU) of *S. epidermidis* and *E. coli* grown on agar plates inoculated with bacteria in contact with the evaluated samples for 4 h. The samples were the following: PDMS with an Ag layer modified at the deposition time of 300 s and different currents of 10, 40, and 80 mA, samples with a C/Ag layer combination and subsequent temperature treatment or eventually laser modification and pristine PDMS samples were tested. The prepared samples were compared with the control.

The antibacterial activity of the prepared materials was evaluated by a drop test using two types of model strains of bacteria: Gram-negative bacteria *Escherichia coli* (*E. coli*) and Gram-positive *Staphylococcus epidermidis* (*S. epidermidis*). The antibacterial activity was assessed after 1 and 4 h in contact with the evaluated samples. The results in Figure 9 show the number of colony-forming units (CFU) of *S. epidermidis* and *E. coli* grown on agar plates inoculated by bacterial samples applied to the surface for 4 h. PDMS samples with an Ag layer at the deposition time of 300 s and different currents of 10, 40, and 80 mA, samples with a C/Ag layer combination, and subsequent temperature treatment or eventually laser modification and pristine PDMS samples were tested. The prepared samples were compared with the control (bacteria incubated in PBS).

Figure 9 shows the evaluated data of the performed antibacterial experiments. With increasing current used, and thus the amount of Ag in the sample, a more potent antibacterial effect can be observed, both for *E. coli* and *S. epidermidis*. A particular antibacterial effect has already been shown on an Ag sample (10 mA, 300 s), i.e., the sample with the lowest current value; this sample shows the thinnest layer of silver. After 4 h, the number of CFU decreased compared to the control. The explanation of good antibacterial properties of pristine against *S. epidermidis* is that PDMS possess hydrophobic properties which inhibited the bacteria growth. It is possible to evaluate the substantial bactericidal effect of an Ag sample (80 mA, 300 s) for both bacterial strains. Therefore, it can be stated that Ag nanolayers deposited on PDMS show strong antibacterial effects against both Gram-positive (*S. epidermidis*) and Gram-negative bacteria (*E. coli*) even when very thin layers of Ag are applied (as discussed in the UV-VIS section). To determine and compare the antibacterial activity, we tested the samples in a combination of C with Ag layers subsequently modified with increased temperature and laser exposure. Promising results against gram-positive and gram-negative bacteria were observed for silver nanocomposites with nanoparticles prepared by bio-reduction method [50], while Ag nanoparticles were also recently prepared by a “green” approach in eggshell powder [51].

**Figure 10:**
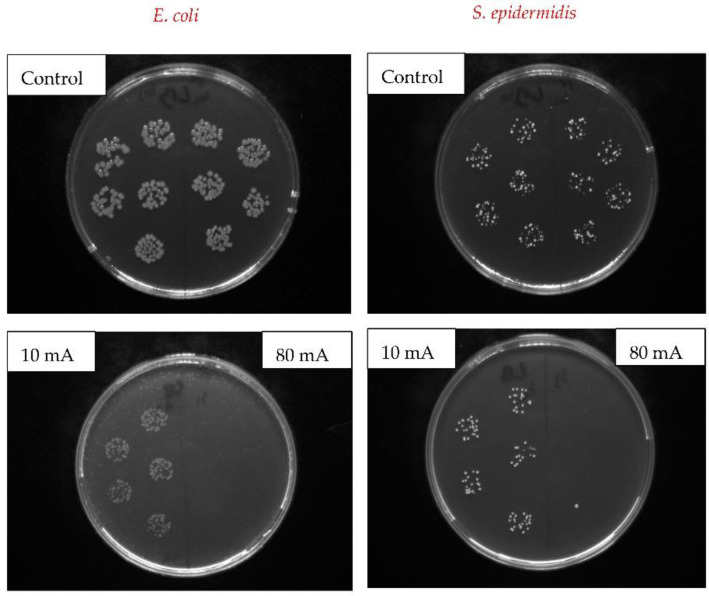
Images of Petri dishes with grown colony-forming units of *E. coli* and *S. epidermidis* on control samples in comparison to samples sputtered with Ag for 400 s at 10 and 80 mA current.

As can be seen from Figure 9, the C/Ag sample exhibited very similar antibacterial properties as the sample without a C layer. The reason may be the order of the deposited layers, where the C layer was located below the Ag layer so that the bacteria competed only with the Ag surface. However, the antibacterial activity decreased after heating the sample (C/Ag/T) above the PDMS operating temperature. This could be due to the ability of the penetrating Ag nanoparticles into the volume of the polymer through the C layer, which reduced the concentration of Ag atoms on the surface. On the other hand, after exposing the samples to the laser beam (C/Ag/T/L), a surface with great antibacterial activity was created again for both bacterial strains. For a better idea of the inhibition of bacterial growth on the surface of the prepared samples, images were obtained after incubation of *S. epidermidis* and *E. coli* for 4-h incubation (see Figure 10). In the images, samples with a sputtered Ag layer (400 s and 10 or 80 mA) are compared with a control sample. As can be seen from Figure 10, after sputtering a thick layer of Ag (80 mA), the bacteria did not survive on the surface.

The mechanism of the antibacterial activity of nanostructured Ag has not been fully clarified yet. However, it has been found that the mechanism consists of two synergistic processes: direct contact of the bacterial cell with metallic Ag on the one hand, and on the other release of Ag^+^ ions into the surrounding medium and its subsequent interaction with the cell, depending on the specific case that prevails. Morphology, roughness, and hydrophilicity/hydrophobicity of the surface of the material play a major role in the direct effect, and each of these parameters affects Gram-negative and Gram-positive bacterial strains differently. Ag ions interact with four major components of the bacterial cell: cell wall, plasma membrane, bacterial DNA, and proteins (e.g., specific enzymes involved in vital cellular processes such as the electron transport chain) [52,53]. In our case the antibacterial activity of *E. coli* is caused only by the release of Ag, which is strongly supported by the presence of base carbon layer and subsequent exposure with excimer laser, which seems to be a crucial step for preparation of material with “ideal” antibacterial properties. For *S. epidermidis*, however, a low wettability of the substrate is also an important factor in our case. Ions cause degradation of the peptidoglycan cell wall and cell lysis (cell death), preventing further bacterial proliferation. Subsequently, they penetrate into the inner part of the cell where they bind on the basis of DNA. Chromosomal aberrations caused by Ag^+^ are also a frequent phenomenon. In the interior of the cell, ions can further cause mitochondrial dysfunction and ribosome denaturation, thereby inhibiting protein synthesis and degrading the plasma membrane. This multi-stage mechanism of antibacterial action is a major factor that results in low bacterial resistance to Ag [52,53]. The release rate of Ag/Ag^+^ depends on the chemical form of Ag, particle/cluster size, surface functionalization, and crystallinity and nature of the surrounding medium (the presence of salts or biomolecules) [54]. A comprehensive study of antibacterial activity of colloidal silver against Gram-negative and Gram-positive bacteria was made in [55], and the results suggest that colloidal silver (CS) could be an effective treatment for infections caused by MDR Gram-negative and Gram-positive bacteria.

## 4. Conclusions

Here, we were able to prepare silver-coated flexible polymer with a surface plasmon resonance effect, the optical properties of which were further enhanced by the heat treatment procedure. By a combination of different elemental analyses (XPS and EDS technique), we have determined that, mostly during the heat treatment process, the surface of PDMS-Ag composite with a wrinkle-like pattern was formed, but the surface plasmon resonance was still maintained. The primary carbon nanolayer was further sputtered as a basic layer affecting the surface morphology for those samples which were subsequently treated with excimer laser. Plasmon resonance effect was determined for an as-sputtered and heat-treated Ag on polydimethylsiloxane. We have implemented sputtering of the carbon base layer prior to Ag sputtering in combination with following excimer laser exposure of PDMS/C/Ag structure to modify its properties, with the aim of antibacterial improvement. We have confirmed that deposition of primary carbon layer on PDMS, followed by sputtering of silver combined with modification of such surface with heat treatment and excimer laser exposure, exhibited strong antibacterial properties against two bacterial strains of *S. epidermidis* and *E. coli*.

## Figures and Tables

**Table 1:** Surface free energy of PDMS foils deposited with Ag and current 20 mA and the same set of samples which underwent heat treatment (285 °C, 1 h).

Deposition Time [s]	50	100	200	400	1000
Surface energy [mJ·m^−2^] - as sputtered	29.7	11.3	14.5	7.3	7.8
Surface energy [mJ·m^−2^] sputtered + heat treated	8.0	13.0	11.5	11.0	18.2

**Table 2:** Positions of absorption maxima of individual samples.

Time [s]	20 mA	20 mA_T	40 mA
	λ_max_ [nm]	λ_max_ [nm]	λ_max_ [nm]
50 s	448	418	424
100 s	430	436	420
200 s	422	450	421
400 s	432	447	406
1000 s	432	437	456

## Data Availability

Data is contained within the article.

## References

[1] Olayil R., Arumugaprabu V., Das O., Lenin Anselm W.A. (2021). A Brief Review on Effect of Nano fillers on Performance of Composites. IOP Conf. Ser. Mater. Sci. Eng..

[2] Kvítek O., Kopáček V., Řezníčková A., Švorčík V. (2018). Detection of organic vapors on sputtered and annealed thin Au film. J. Phys. Conf. Ser..

[3] Crosby A.J., Lee J.Y. (2007). Polymer Nanocomposites: The “Nano” Effect on Mechanical Properties. Polym. Rev..

[4] Alexandre M., Dubois P. (2000). Polymer-layered silicate nanocomposites: Preparation, properties and uses of a new class of materials. Mater. Sci. Eng. R Rep..

[5] Mousavi S.R., Estaji S., Kiaei H., Mansourian-Tabaei M., Nouranian S., Jafari S.H., Ruckdaschel H., Arjmand M., Khonakda H.A. (2022). A review of electrical and thermal conductivities of epoxy resin systems reinforced with carbon nanotubes and graphene-based nanoparticles. Polym. Test..

[6] Geim A.K., Novoselov K.S. (2007). The rise of graphene. Nat. Mater.

[7] Novoselov K.S., Geim A.K., Morozov S.V., Jiang D., Zhang Y., Dubonos S.V., Grigorieva I.V., Firsov A.A. (2004). Electric Field Effect in Atomically Thin Carbon Films. Science.

[8] Al Sheheri S.Z., Al-Amshany Z.M., Al Sulami Q.A., Tashkandi N.Y., Hussein M.A., El-Shishtawy R.M. (2019). The preparation of carbon nanofillers and their role on the performance of variable polymer nanocomposites. Des. Monomers Polym..

[9] Zhang F., Yang K., Liu G., Chen Y., Wang M., Li S., Li R. (2022). Recent advances on graphene: Synthesis, properties and applications. Compos. Part A.

[10] Wei J., Atif R., Vo T., Inam F. (2015). Graphene Nanoplatelets in Epoxy System: Dispersion, Reaggregation, and Mechanical Properties of Nanocomposites. J. Nanomater..

[11] Esfandiari M., Lalbakhsh A., Shehni P.N., Jarchi S., Ghaffari-Miab M., Mahtaj H.N., Reisenfeld S., Alibakhshikenari M., Koziel S., Szczepanski S. (2022). Recent and emerging applications of Graphene-based metamaterials in electromagnetics. Mater. Des..

[12] Yavari F., Koratkar N. (2012). Graphene-Based Chemical Sensors. J. Phys. Chem. Lett..

[13] Šupová M., Martynková G.S., Barabaszová K. (2011). Effect of nanofillers dispersion in polymer matrices: A review. Sci. Adv. Mater..

[14] Rajak D.K., Pagar D.D., Menezes P.L., Linul E. (2019). Fiber-Reinforced Polymer Composites: Manufacturing, Properties, and Applications. Polymers.

[15] Szeluga U., Kumanek B., Trzebicka B. (2015). Synergy in hybrid polymer/nanocarbon composites. A review. Compos. Part A Appl. Sci. Manuf..

[16] Merino C.A.I., Ledezma Sillas J.E., Mezaa J.M., Herrera Ramirez J.M. (2017). Metal matrix composites reinforced with carbon nanotubes by an alternative technique. J. Alloys Compd..

[17] Shit S.C., Shah P. (2013). A Review on Silicone Rubber. Natl. Acad. Sci. Lett..

[18] Eduok U., Faye O., Szpunar J. (2017). Recent developments and applications of protective silicone coatings: A review of PDMS functional materials. Prog. Org. Coat..

[19] Li X., Gao Z., Li B., Zhang X., Li Y., Sun J. (2021). Self-healing superhydrophobic conductive coatings for self-cleaning and humidity-insensitive hydrogen sensors. Chem. Eng. J..

[20] Razavi M., Primavera R., Vykunta A., Thakor A.S. (2021). Silicone-based bioscaffolds for cellular therapies. Mater. Sci. Eng. C.

[21] Pino C.J., Haselton F.R., Chang M.S. (2005). Seeding of Corneal Wounds by Epithelial Cell Transfer from Micropatterned PDMS Contact Lenses. Cell Transplant..

[22] Rus D., Tolley M.T. (2015). Design, fabrication and control of soft robots. Nature.

[23] Burda C., Chen X., Narayanan R., El-Sayed M.A. (2005). Chemistry and Properties of Nanocrystals of Different Shapes. Chem. Rev..

[24] El-Sayed M.A. (2001). Some Interesting Properties of Metals Confined in Time and Nanometer Space of Different Shapes. Acc. Chem. Res..

[25] Sershen S.R., Westcott S.L., Halas N.J., West J.L. (2000). Temperature-sensitive polymer–nanoshell composites for photothermally modulated drug delivery. J. Biomed. Mater. Res..

[26] Cobley C.M., Chen J., Cho E.C., Wang L.V., Xia Y. (2011). Gold nanostructures: A class of multifunctional materials for biomedical applications. Chem. Soc. Rev..

[27] Qureshia D., Nayak S.K., Maji S., Anis A., Kim D., Pal K. (2019). Environment sensitive hydrogels for drug delivery applications. Eur. Polym. J..

[28] McGillicuddy E., Murray I., Kavanagh S., Morrison L., Fogarty A., Cormican M., Dockery P., Prendergast M., Rowan N., Morris D. (2017). Silver nanoparticles in the environment: Sources, detection and ecotoxicology. Sci. Total Environ..

[29] Gherasim O., Puiu R.A., Bîrcă A.C., Burdușel A.-C., Grumezescu A.M. (2020). An Updated Review on Silver Nanoparticles in Biomedicine. Nanomaterials.

[30] Alonso J.C., Diamant R., Castillo P., Acosta–García M.C., Batina N., Haro-Poniatowski E. (2009). Thin films of silver nanoparticles deposited in vacuum by pulsed laser ablation using a YAG:Nd laser. Appl. Surf. Sci..

[31] Cao C., Zhang T., Yang N., Niu X., Zhou Z., Wang J., Yang D., Chen P., Zhong L., Dong X. (2022). POD Nanozyme optimized by charge separation engineering for light/pH activated bacteria catalytic/photodynamic therapy. Signal Transduct. Target. Ther..

[32] Kelly P.J., Arnell R.D. (2000). Magnetron sputtering: A review of recent developments and applications. Vacuum.

[33] Chu C., Hu X., Yan H., Sun Y. (2021). Surface functionalization of nanostructured Cu/Ag-deposited polypropylene fiber by magnetron sputtering. e-Polymers.

[34] Garrett T.R., Bhakoo M., Zhang Z. (2008). Bacterial adhesion and biofilms on surfaces. Prog. Nat. Sci..

[35] Song F., Koo H., Ren D. (2015). Effects of Material Properties on Bacterial Adhesion and Biofilm Formation. J. Dent. Res..

[36] Hu H., Burrow M.F., Leung W.K. (2022). Evaluation of 12-hour in situ bacterial colonization on smooth restorative material surfaces. J. Dent..

[37] Mather R.R. (2009). Surface modification of textiles by plasma treatments. Surface Modification of Textiles.

[38] Hurtuková K., Fajstavrová K., Rimpelová S., Vokatá B., Fajstavr D., Slepičková Kasálková N., Siegel J., Švorčík V., Slepička P. (2021). Antibacterial Properties of a Honeycomb-like Pattern with Cellulose Acetate and Silver Nanoparticles. Materials.

[39] Mafuné F., Kohno J.-Y., Takeda Y., Kondow T., Sawabe H. (2000). Formation and Size Control of Silver Nanoparticles by Laser Ablation in Aqueous Solution. J. Phys. Chem. B.

[40] Philip P., Jose T., Philip K.C., Manoj P., Sajini T. (2020). Studies on the hypsochromic shifted optical properties of gold nanoparticles embedded electrospun poly(methyl methacrylate) (PMMA) nanofibers. Mater. Today Proc..

[41] Liu X., Li D., Sun X., Li Z., Song H., Jiang H., Chen Y. (2015). Tunable Dipole Surface Plasmon Resonances of Silver Nanoparticles by Cladding Dielectric Layers. Sci. Rep..

[42] Shivashankar H., Kevin A.M., Manohar S.B.S., Kulkarni S.M. (2021). Investigation on dielectric properties of PDMS based nanocomposites. Phys. B Condens. Matter.

[43] Ferreira T.P.M., Nepomuceno N.C., Medeiros E.L.G., Medeiros E.S., Sampaio F.C., Oliveira J.E., Oliveira M.P., Galvão L.S., Bulhões E.O., Santos A.S.F. (2019). Antimicrobial coatings based on poly(dimethyl siloxane) and silver nanoparticles by solution blow spraying. Prog. Org. Coat..

[44] Slepička P., Hurtuková K., Fajstavr D., Slepičková Kasálková N., Lyutakov O., Švorčík V. (2021). Carbon-gold nanocomposite induced by unique high energy laser single-shot annealing. Mater. Lett..

[45] Jing Y., Wang R., Wang. Q., Xiang Z., Li Z., Gu H., Wang X. (2021). An overview of surface-enhanced Raman scattering substrates by pulsed laser deposition technique: Fundamentals and applications. Adv. Compos. Hybrid Mater..

[46] Kima J.H., Twaddle K.M., Cermak L.M., Jang W., Yun J., Byun H. (2016). Photothermal heating property of gold nanoparticle loaded substratesand their SERS response. Colloids Surf. A Physicochem. Eng. Asp..

[47] Slepičková Kasálková N., Slepička P., Švorčík V. (2021). Carbon Nanostructures, Nanolayers, and Their Composites. Nanomaterials.

[48] Lišková J., Slepičková Kasálková N., Slepička P., Švorčík V., Bačáková L. (2019). Heat-treated carbon coatings on poly (L-lactide) foils for tissue engineering. Mater. Sci. Eng. C.

[49] Slepicka P., Siegel J., Lyutakov O., Slepickova Kasalkova N., Kolska Z., Bacakova L., Svorcik V. (2018). Polymer nanostructures for bioapplications induced by laser treatment. Biotechnol. Adv..

[50] Deeksha B., Sadanand V., Hariram N., Rajulu A.V. (2021). Preparation and properties of cellulose nanocomposite fabrics with in situ generated silver nanoparticles by bioreduction method. J. Bioresour. Bioprod..

[51] Yorseng K., Siengchin S., Ashok B., Rajulu A.V. (2020). Nanocomposite egg shell powder with in situ generated silver nanoparticles using inherent collagen as reducing agent. J. Bioresour. Bioprod..

[52] Polívková M., Hubáček T., Staszek M., Švorčík V., Siegel J. (2017). Antimicrobial Treatment of Polymeric Medical Devices by Silver Nanomaterials and Related Technology. Int. J. Mol. Sci..

[53] Chaloupka K., Malam Y., Seifalian A.M. (2010). Nanosilver as a new generation of nanoproduct in biomedical applications. Trends Biotechnol..

[54] Kittler S., Greulich C., Diendorf J., Koöller M., Epple M. (2010). Toxicity of Silver Nanoparticles Increases during Storage Because of Slow Dissolution under Release of Silver Ions. Chem. Mater..

[55] Domínguez A., Algaba R.A., Canturri A.M., Villodres Á.R., Smani Y. (2020). Antibacterial Activity of Colloidal Silver against Gram-Negative and Gram-Positive Bacteria. Antibiotics.

